# 小于1 cm的肺实性结节区分肺癌与肺内淋巴结的多因素分析

**DOI:** 10.3779/j.issn.1009-3419.2021.102.05

**Published:** 2021-02-20

**Authors:** 继征 汤, 春全 刘, 沛豪 王, 永 崔

**Affiliations:** 100050 北京，首都医科大学附属北京友谊医院胸外科 Department of Thoracic Surgery, Beijing Friendship Hospital of Capital Medical University, Beijing 100050, China

**Keywords:** 肺实性结节, 肺内淋巴结, 肺恶性结节, 受试者工作曲线, Solid pulmonary nodules, Intrapulmonary lymph nodes, Malignant pulmonary nodules, Receiver operating characteristic (ROC) curve

## Abstract

**背景与目的:**

肺实性小结节的术前诊断及鉴别诊断十分困难。计算机断层扫描（computed tomography, CT）作为肺癌筛查的常用手段，被广泛应用于临床。本研究旨在对 < 1 cm的肺实性结节临床诊疗中肺恶性结节与肺内淋巴结患者的临床资料进行分析，为两者的鉴别提供参考。

**方法:**

回顾性分析2017年6月-2020年6月行手术治疗的肺实性结节患者。共收集了145个结节（肺腺癌60个，肺类癌2个，恶性间皮瘤1个，肉瘤样癌1个，淋巴结81个）的患者临床资料，最终分为肺腺癌和肺内淋巴结两组，并对其临床资料进行了统计分析。根据单因素分析（*χ*^2^检验、*t*检验）结果筛选有统计学差异的变量，纳入Logistic回归多因素分析，确定预测变量并绘制受试者工作曲线（receiver operating characteristic, ROC）曲线，得到曲线下面积（area under the curve, AUC）值。

**结果:**

*Logistic*回归分析显示结节最长径、Max CT值、分叶征和毛刺征是肺腺癌与肺内淋巴结鉴别的重要指标，风险比分别为106.645（95%CI: 3.828-2, 971.220, *P* < 0.01）、0.980（95%CI: 0.969-0.991, *P* < 0.01）、3.550（95%CI: 1.299-9.701, *P*=0.01）、3.618（95%CI: 1.288-10.163, *P*=0.02）。根据Logistic回归分析结果确定预测模型，绘制ROC曲线，计算曲线下面积AUC值=0.877（95%CI: 0.821-0.933, *P* < 0.01）。

**结论:**

对于 < 1 cm的肺实性结节，在众多因素中，肺结节最长径、Max CT值、分叶征和毛刺征对鉴别肺恶性结节和肺内淋巴结更为重要。

## Abstract (en)

**Background and objective:**

Preoperative diagnosis and differential diagnosis of small solid pulmonary nodules are very difficult. Computed tomography (CT), as a common method for lung cancer screening, is widely used in clinical practice. The aim of this study was to analyze the clinical data of patients with malignant pulmonary nodules and intrapulmonary lymph nodes in the clinical diagnosis and treatment of < 1 cm solid pulmonary nodules, so as to provide reference for the differentiation of the two.

**Methods:**

Patients with solid pulmonary nodules who underwent surgery from June 2017 to June 2020 were analyzed retrospectively. The clinical data of 145 nodules (lung adenocarcinoma 60, lung carcinoid 2, malignant mesothelioma 1, sarcomatoid carcinoma 1, lymph node 81) were collected and finally divided into two groups: lung adenocarcinoma and intrapulmonary lymph nodes, and their clinical data were statistically analyzed. According to the results of univariate analysis (*χ*^2^ test, *t* test), the variables with statistical differences were selected and included in *Logistic* regression multivariate analysis. The predictive variables were determined and the receiver operating characteristic (ROC) curve was drawn to get the area under the curve (AUC) value of the area under the curve.

**Results:**

*Logistic* regression analysis showed that the longest diameter, Max CT value, lobulation sign and spiculation sign were important indicators for distinguishing lung adenocarcinoma from intrapulmonary lymph nodes, and the risk ratios were 106.645 (95%CI: 3.828-2, 971.220, *P* < 0.01), 0.980 (95%CI: 0.969-0.991, *P* < 0.01), 3.550 (95%CI: 1.299-9.701, *P*=0.01), 3.618 (95%CI: 1.288-10.163, *P*=0.02). According to the results of *Logistic* regression analysis, the prediction model is determined, the ROC curve is drawn, and the AUC value under the curve is calculated to be 0.877 (95%CI: 0.821-0.933, *P* < 0.01).

**Conclusion:**

For < 1 cm solid pulmonary nodules, among many factors, the longest diameter, Max CT value, lobulation sign and spiculation sign are more important in distinguishing malignant pulmonary nodules from intrapulmonary lymph nodes.

肺癌发病率和死亡率均为恶性肿瘤的首位，是癌症相关死亡的最主要原因^[1, 2]^。计算机断层扫描（computed tomography, CT）作为肺癌筛查的常用手段，被广泛应用于临床^[3, 4]^。根据肺结节密度的不同，分为实性结节和亚实性结节（包括磨玻璃结节和部分实性结节）^[5]^。有研究^[6-9]^显示，在肺癌的CT筛查研究中，肺实性结节的恶性率高于亚实性结节，且恶性肺实性结节倍增时间短，恶性程度高，转移发生早，预后更差。因此，肺实性结节的早期诊断和治疗尤为重要。

肺结节影像学特征，如分叶、毛刺等与恶性结节密切相关^[10-13]^，可作为临床诊疗中区分良恶性的重要依据，然而，这些特征在 < 1 cm的肺实性结节中表现并不明显。CT随访可减少良性结节进行不必要的手术，但随访会延误肺恶性肿瘤的诊断和治疗^[14]^。综上，鉴别肺恶性结节和肺内淋巴结十分困难。本研究的目的是根据临床资料（CT特征、家族史、吸烟史、年龄、性别、肿瘤标志物等），为临床中鉴别肺恶性结节和肺内淋巴结提供参考。

## 1 资料与方法

### 1.1 一般资料

收集本院在2017年6月-2020年6月期间行亚肺叶切除或肺叶切除术的患者。所有患者均在手术前接受了全面的术前检查。纳入标准：①最长径 < 1 cm的实性结节；②胸部CT扫描与手术之间的间隔为1个月之内；③病理为淋巴结或肺恶性结节。排除标准：①CT图像质量较差；②临床资料不全。该研究共包括145个结节。

### 1.2 图像分析方法

所有患者均采用GE Revolution 256排螺旋CT机，将双手放在头部附近，仰卧。从胸腔入口水平到肺底部以下进行图像采集。扫描方式：管电压为120 kV，自动管电流，螺距0.984:1，旋转时间0.5 s，图像层厚1.25 mm，层间距1.25 mm。通过纵隔窗口（宽度400 HU；水平40 HU）和肺部窗口（宽度1, 600 HU；水平-700 HU）设置获得图像。两名主任医师职称胸部放射科医生检查了所有患者的CT数据，他们对病变的病理结果不知情。解释上的差异（如有）已通过协商解决。在CT图像上评估了病变的以下特征：结节最长径、结节是否分叶、结节边缘是否存在毛刺、肺窗中结节的Max CT值。

### 1.3 统计学方法

根据病理类型将淋巴结和肺恶性结节分为两组，在收集数据的过程中，共收集了除肺腺癌外的其他肺恶性肿瘤4例，其中恶性间皮瘤1例，肉瘤样癌1例，肺类癌2例，由于例数较少，未纳入统计学分析。对每组的临床资料进行统计学分析。连续变量表示为平均值±标准偏差，而分类变量表示为绝对数。使用独立样本*t*检验比较不同组之间的年龄、结节大小、癌胚抗原（carcinoembryonic antigen, CEA）、神经元特异性烯醇化酶（neuron-specific enolase, NSE）、可溶性细胞角蛋白19片段（cytokeratin 19 fragment, CYFRA21-1）、胃泌素释放肽前体（pro-gastrin releasing peptide, ProGRP）、Max CT值。*χ*^2^检验用于比较各组患者的性别、肺癌家族史、吸烟史、分叶征、毛刺征。二元*Logistic*回归分析用于检验哪些临床资料可以作为肺癌的预测变量。受试者工作特征曲线（receiver operating characteristic curve, ROC），用于计算预测变量对于鉴别肺腺癌与肺内淋巴结的预测值。*P* < 0.05被认为是具有显著统计学差异。所有统计分析均使用统计软件SPSS 22.0进行。

## 2 结果

### 2.1 入组患者临床资料及单因素分析

根据病理类型分组，镜下见淋巴组织伴碳末沉着，考虑为淋巴结的共81例；根据2011年国际肺癌研究协会（International Association for the Study of Lung Cancer, IASLC）/美国胸科学会（American Thoracic Society, ATS）/欧洲呼吸学会（European Respiratory Society, ERS）的肺腺癌分类标准分类^[15]^，60例肺腺癌患者中，有3例不典型腺瘤样增生（atypical adenomatous hyperplasia, AAH），2例原位腺癌（adenocarcinoma *in situ*, AIS），2例微浸润性腺癌（minimally invasive adenocarcinoma, MIA）和53例浸润性腺癌（invasive adenocarcinoma, IAC）

在定量变量中，结节最长径（*P* < 0.001）及Max CT值（*P*=0.028）在肺内淋巴结与肺腺癌间存在统计学差异。在定性变量中，分叶征（*P* < 0.001）及毛刺征（*P* < 0.001）在肺内淋巴结与肺腺癌间存在统计学差异。在两组患者的临床资料中，性别、年龄、吸烟史、肺癌家族史、CEA、NSE、CYFRA211和ProGRP均无显著差异（*P* > 0.05），见表 1及表 2。

**1:** 定量变量以及单因素分析结果 The quantitative variables and the results of univariate analysis

Quantitative variables	Lymph node group (*n*=81) (Mean±SD, range)	Lung adenocarcinoma group (*n*=60) (Mean±SD, range)	*P*
Age (yr)	59.02±9.83 (30-78)	60.80±8.84 (37-81)	0.271
CEA	2.37±1.13 (0.37-5.50)	3.19±3.52 (0.66-26.81)	0.052
NSE	13.98±3.43 (8.91-26.80)	14.55±5.23 (7.71-33.57)	0.440
CYFRA211	2.57±1.05 (0.71-6.02)	2.74±1.31 (0.93-6.6)	0.386
ProGRP	41.51±18.14 (15.18-153.55)	43.49±27.13 (16.17-224.34)	0.604
Size (cm)	0.72±0.16 (0.38-0.99)	0.83±0.15 (0.41-0.99)	< 0.001
Maximum CT value (HU) of ROI	90.43±193.93 (-153-1634)	33.63±45.52 (-174-114)	0.028
CEA: carcinoembryonic antigen; NSE: neuron-specific enolase; CYFRA21-1: cytokeratin 19 fragment; ProGRP: pro-gastrin releasing peptide; CT: computed tomography; ROI: region of interest.			

**2:** 定性变量以及单因素分析结果 The qualitative variables and the results of univariate analysis

Variables		Lymph node group (*n*=81)	Lung adenocarcinoma group (*n*=60)	*P*
Gender	Male	41	28	0.643
	Female	40	32	
Lobulation	Yes	17	44	< 0.001
	No	64	16	
Spiculation	Yes	16	40	< 0.001
	No	65	20	
Smoking status	Yes	27	18	0.675
	No	54	42	
Family history of lung cancer	Yes	1	2	0.393
	No	80	58	

### 2.2 *Logistic*回归分析

根据单因素分析结果将结节最长径，Max CT值、分叶征、毛刺征纳入*Logistic*回归分析。结果显示结节最长径大、Max CT值小、分叶征和毛刺征是肺腺癌与肺内淋巴结鉴别的重要指标，风险比分别为106.645（95%CI: 3.828-2, 971, *P* < 0.01）、0.980（95%CI: 0.969-0.991, *P* < 0.01）、3.550（95%CI: 1.299-9.701, *P*=0.01）、3.618（95%CI: 1.288-10.163, *P*=0.02），见表 3。选取肺内淋巴结及肺腺癌各组中的1个结节，肺内淋巴结结节CT影像表现为：右肺下叶背段胸膜下可见实性结节，其内可见钙化，直径0.72 cm，边缘清晰，其组织病理镜下表现为：淋巴组织伴碳末沉着；肺腺癌结节CT影像表现为：左肺上叶前段见直径0.96 cm实性结节，其内可见分叶，边缘毛糙见毛刺，其组织病理镜下表现为：浸润性肺腺癌改变，腺泡为主型，见图 1。

**3:** 肺腺癌组与淋巴结组二元*Logistic*回归分析结果 The binary *Logistic* regression analysis of lung adenocarcinoma group and lymph node group

Item	OR	95%CI	*P*
Size	106.645	3.828-2, 971	0.006
Maximum CT value of ROI	0.980	0.969-0.991	0.001
Lobulation	3.550	1.299-9.701	0.014
Spiculation	3.618	1.288-10.163	0.015

**1:**
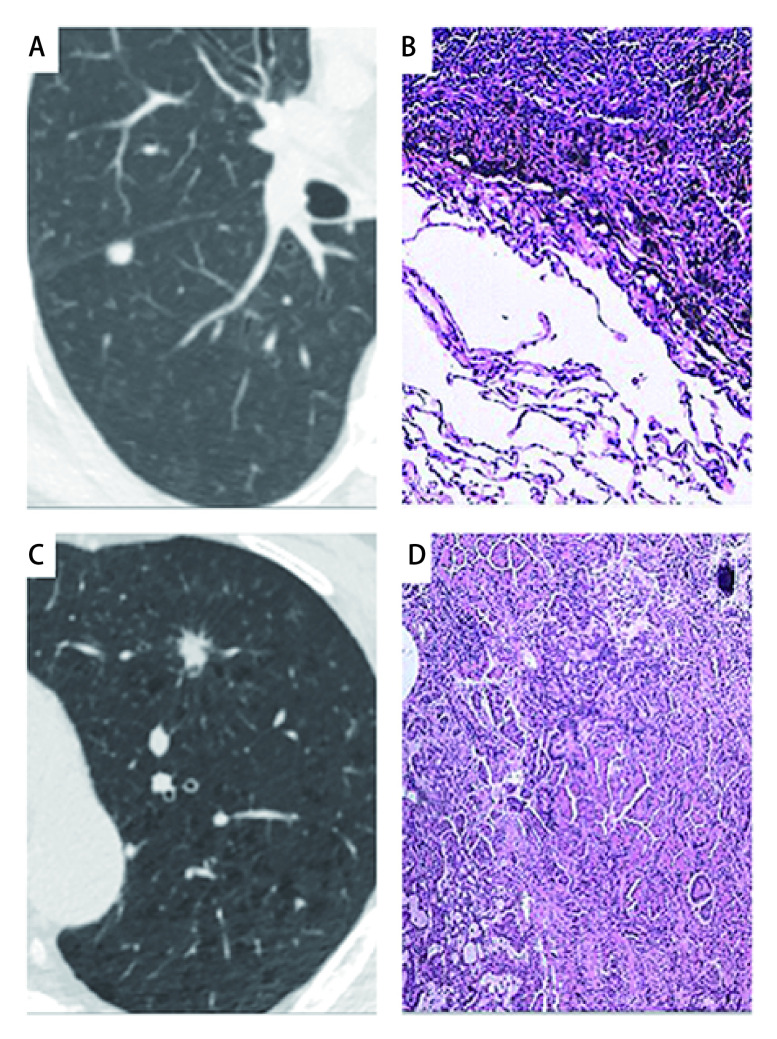
1例肺腺癌结节及1例肺内淋巴结结节的CT影像及组织病理镜下表现。A：女性，66岁，实性结节（0.72 cm），无分叶及毛刺；B：病理结果为淋巴结（HE，×400）；C：男性，60岁，实性结节（0.96 cm），分叶状，毛刺征；D：病理结果为浸润性腺癌（HE，×400）。 CT images and histopathological findings of 1 case of pulmonary adenocarcinoma nodule and 1 case of pulmonary lymph node nodule. A: Female, 66 years old, a solid nodule (0.72 cm), no lobulation and speculation; B: pathology: lymph node (HE, ×400); C: Male, 60 years old, a solid nodule (0.96 cm), lobulation, speculation; D: pathology: invasive adenocarcinoma (HE, ×400).

### 2.3 预测模型的建立及其诊断效能

根据*Logistic*回归分析结果，将结节大小、Max CT值、分叶征、毛刺征作为肺腺癌的预测变量，绘制ROC曲线，计算曲线下面积（area under the curve, AUC）值=0.877（95%CI: 0.821-0.933），灵敏度为76.7%，特异度为85.2%，见图 2。

**2:**
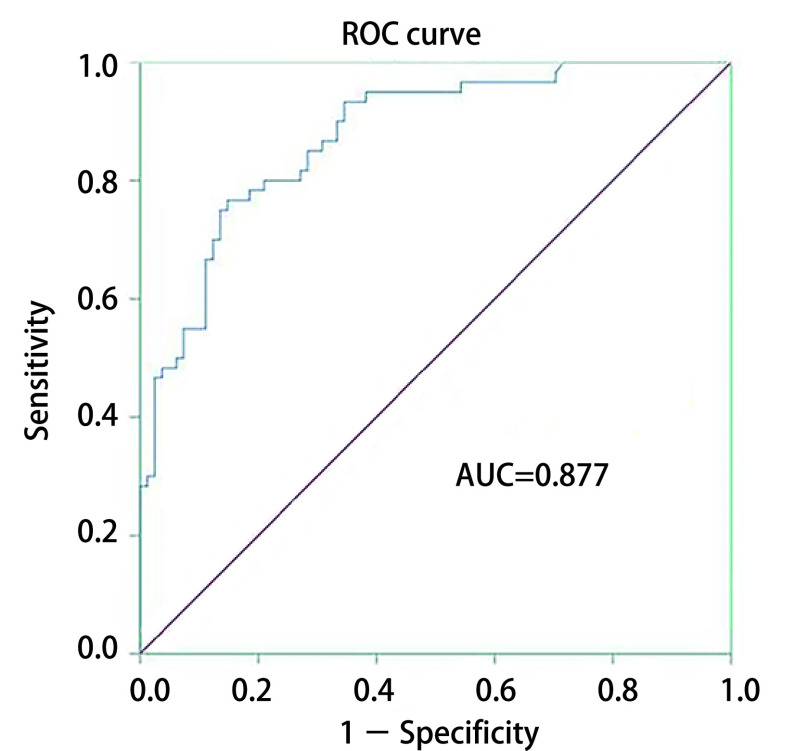
预测模型的ROC曲线。预测模型的AUC值为0.877。 The ROC curves for the prediction model. The AUC value using the prediction model was 0.877. ROC: receiver operating characteristic; AUC: area under the curve.

## 3 讨论

随着CT技术的发展及应用，越来越多的肺结节被发现，对于肺结节的处理众多指南层出不穷，但对于肺结节良恶性的鉴别，仍然存在很多问题。 < 1 cm的肺实性结节的CT形态学特征不明显，非手术活检很难明确诊断，因此，如何鉴别具有分叶或毛刺状外观的肺内淋巴结和实体肺癌仍然是一个挑战。临床中诊断和治疗肺实性小结节的常用方法为手术切除、非手术活检和CT随访，每种方法都有其各自的优缺点，手术切除病灶行病理诊断是金标准，是恶性结节的有效治疗方法，但对于良性结节来说应尽量避免手术；非手术活检具有侵入性，操作具有潜在风险，并且经常无法明确诊断。

本研究共收集了145个结节的患者临床资料，根据最终病理类型分为淋巴结组和肺腺癌组，其中淋巴结81例；肺腺癌组中AAH 2例（5.0%），AIS 2例（3.3%），MIA 3例（3.3%），IAC 53例（88.3%），在诊断为肺腺癌的实性小结节患者中，大多数为浸润性腺癌，恶性度较高，发展较快，所以术前根据临床资料，区分肺内淋巴结和肺腺癌对患者的管理具有重要意义。我们的研究表明，两组患者的部分影像学临床资料有明显的统计学差异，其余临床资料对区分肺内淋巴结和肺癌的预测价值不明显。最终纳入预测模型的临床资料有结节最长径，Max CT值，分叶征和毛刺征，预测模型的总体准确性为87.7%。其中结节最长径的风险比高达106.645，根据统计分析结果，在 < 1 cm的实性结节中，结节最长径越大，其为肺腺癌，而不是淋巴结的概率越高。而其他研究表明，在CT初筛中预测肺恶性肿瘤最常发生在实性结节最长径 > 6 mm时^[4, 16]^，随着结节最长径的增加，结节被预测为恶性肿瘤的概率随之增加，并且实性结节最长径 < 6 mm时仅有0.3%被预测为恶性肿瘤^[4]^。分叶征、毛刺征提示恶性肿瘤已被多项研究报道证实，尽管有研究表明，与较大的肺实性结节相比，6 mm-15 mm的肺实性结节的影像学特征，如毛刺征和分叶征的特异性较低，良、恶性结节之间的特征有较大的重叠^[16-18]^。但本研究中分叶征、毛刺征仍是鉴别肺腺癌与淋巴结的重要预测变量。目前关于Max CT值预测肺实性结节良恶性的报道较少，本研究发现，Max CT值对于鉴别肺腺癌与肺内淋巴结有显著相关性，随着Max CT值的降低，预测为肺腺癌的概率升高。

综合应用结节最长径、Max CT值、分叶征、毛刺征建立的预测模型，AUC值为0.877，说明了本研究中的预测模型具有良好的预测效能。对于 < 1 cm的肺实性结节，术前难以区分肺腺癌与肺内淋巴结，我们的预测模型具有重要的参考价值。我们的研究目前存在一些局限性，首先我们只入组了行手术治疗的患者，未纳入非手术活检的患者，可能会发生选择偏倚。其次，本研究样本量较小，需要对大量病例进行进一步的前瞻性研究，以验证目前的预测模型。
